# The bionomics of *Anopheles merus* (Diptera: Culicidae) along the Kenyan coast

**DOI:** 10.1186/1756-3305-6-37

**Published:** 2013-02-14

**Authors:** Pamela C Kipyab, Battan M Khaemba, Joseph M Mwangangi, Charles M Mbogo

**Affiliations:** 1 Kenya Medical Research Institute, Center for Geographic Medicine Research-Coast, Kilifi, Kenya; 2 Moi University, Eldoret, Kenya

## Abstract

**Background:**

*Anopheles merus*, a sibling species of the *Anopheles gambiae* complex occurs along the East African coast but its biology and role in malaria transmission in this region is poorly understood. We evaluated the blood feeding pattern and the role of this species in malaria transmission in Malindi district, Coastal Kenya.

**Methods:**

Adult mosquitoes were collected indoors by CDC light traps and Pyrethrum Spray Catch and outdoors by CDC light traps. *Anopheles* females were identified to species by morphological characteristics and sibling species of *An*. *gambiae* complex distinguished by rDNA polymerase chain reaction (PCR). Screening for host blood meal sources and presence or absence of *Plasmodium falciparum* circumsporozoite proteins was achieved by Enzyme Linked Immunosorbent Assays (ELISA).

**Results:**

*Anopheles merus* comprised 77.8% of the 387 *Anopheles gambiae s*.*l* adults that were collected. Other sibling species of *Anopheles gambiae s*.*l* identified in the study site included *An*. *arabiensis*(3.6%), and *An*. *gambiae s*.*s*. (8%). The human blood index for *An*. *merus* was 0.12, while the sporozoite rate was 0.3%.

**Conclusion:**

These findings suggest that *An*. *merus* can play a minor role in malaria transmission along the Kenyan Coast and should be a target for vector control which in turn could be applied in designing and implementing mosquito control programmes targeting marsh-breeding mosquitoes; with the ultimate goal being to reduce the transmission of malaria associated with these vectors.

## Background

In Kenya, three members of the *An*. *gambiae* complex are responsible for malaria transmission including *An*. *gambiae s*.*s*, *An*. *arabiensis* and *An*. *merus*[1-3]. The inability to morphologically distinguish between the three sibling species complicates the planning and maintenance of vector control activities in areas where these species co-occur [4,5], because they differ in their ecology and behaviour and contribute asymmetrically to malaria transmission. Proper identification of these species in a particular region can pave way for meaningful studies on their breeding, oviposition, biting, resting and feeding behaviour all of which are essential for successful management of these species.

In the African continent, *An*. *merus* is exclusively confined to the Eastern coast stretching from South Africa to the horn of Africa [6]. Existing data suggests that this species is mainly zoophilic [7]. Analysis of blood meal sources from anopheline mosquitoes collected along the coast of Kenya showed that *An*. *merus* predominantly fed on humans, suggesting that this species was highly anthropophilic at the coast [8]. In Madagascar, [9], similar observations were recorded. This is in contrast to earlier studies [7] which showed that *An*. *merus* was mainly zoophilic, with a stronger tendency to bite more when outdoors than indoors. Peak biting activities of *An*. *merus* occur between 2400 hours and 0100 hours [7]. Previously *An*. *merus* was considered to play a minor role in malaria transmission [7], but recent studies in Madagascar and coastal parts of Tanzania have elevated the vector status of this species [9,10].

However, no studies have been conducted to elucidate the extent of its efficiency as a vector including its breeding, resting and feeding behaviour. Along the Kenyan coast, there is no recent detailed information on the biology, behaviour and importance of this species as an important vector of malaria. The overall goal of the studies in this paper was to examine the ecology and behaviour of *An*. *merus* and its role in malaria parasite transmission in Garithe area of Malindi District along the Kenyan coast.

## Methods

### Study site

The study was carried out in Malindi district, which is the tenth largest town in Kenya and a major tourist destination in Kilifi County along the Kenya Coast. Malindi town is approximately 108 km north of Mombasa. Entomological sampling was carried out in Garithe village located 27 kilometres north of Malindi town in Kenya. Garithe has been previously described [8,11]. The coastal part of Garithe consists of mangrove trees and the area experiences high tides every month leaving pools of water during the low tides. These pools of salty water provide suitable habitats for *An*. *merus* breeding. The area also has numerous pockets of man-made ponds.

### Entomological sampling

Mosquitoes were collected inside and outside selected homesteads. For indoor collection, mosquitoes were collected weekly in 16 randomly selected houses by use of pyrethrum spray collection method (PSC) [12]. The collections were conducted between 0700 hrs and 1000 hrs from September 2007 to March 2008. Knocked-down mosquitoes were placed in petri-dishes and transported to the KEMRI laboratory for further analysis. Additionally, indoor biting mosquitoes were collected by use of CDC light traps placed inside houses set between 1800 h and 0600 h. Ten traps were set inside 10 selected houses. Outdoor biting mosquitoes were collected by the use of CDC light traps with a lid hung outside five selected houses and cattle sheds.

### Laboratory processing of mosquitoes

In the laboratory, female *Anopheles* were morphologically identified to species [13]. The legs of each *Anopheles gambiae s*.*l*. were detached from the rest of the body and used for sibling species identification using rDNA-PCR [4]. The heads and thorax were used for *Plasmodium falciparum* sporozoite Enzyme Linked Immunosorbent Assay (ELISA) [14]. The fully blood fed abdomens were tested for host sources of blood by ELISA [15]. Test samples were visually assessed for positivity [16].

### Entomological indices

Entomological Inoculation rates (EIR), a standard measure of transmission intensity, is expressed as the number of infective bites per person per unit time (e.g., daily, monthly, yearly). The EIR was obtained by multiplying the human-biting rate by the proportion of sporozoite positive mosquitoes. The human blood index was determined as the proportion of blood-fed mosquitoes that had fed on humans out of the total number tested. The feeding success was determined as the proportion of blood fed and semi-gravid mosquitoes in the total proportion presumed to have been trying to feed (all mosquitoes except gravids) [17]. The sporozoite index for a given species was calculated as the proportion of females carrying infective sporozoites in the head-thorax. The human-biting rates (the number of biting mosquitoes per human-night), was calculated by dividing the total number of blood-fed and half-gravid mosquitoes caught in PSC catches by the number of persons sleeping in the house the night preceding the collection.

### Statistical analysis

Fisher’s analysis of variance was used to determine the resting and feeding behaviour of *An*. *merus* adults. The data was analysed by the use of SPSS for Windows (version. 15) (CDC Atlanta, USA).

### Ethical considerations

This project involves very minimal interaction with human subjects; therefore no ethical approval was needed.

## Results

*An*. *gambiae s*.*l* represented 96.8% of the total anophelines (n = 400) collected during the six month sampling period. Other species included *An*. *funestus* (2.5%) and *An*. *coustani* (0.8%). The majority of *An*. *gambiae s*.*l* (97.2%) were collected during the short rains between September and December.

### Proportion of *An*. *gambiae s*.*l* adult sibling species found in Garithe

**Figure 1:**
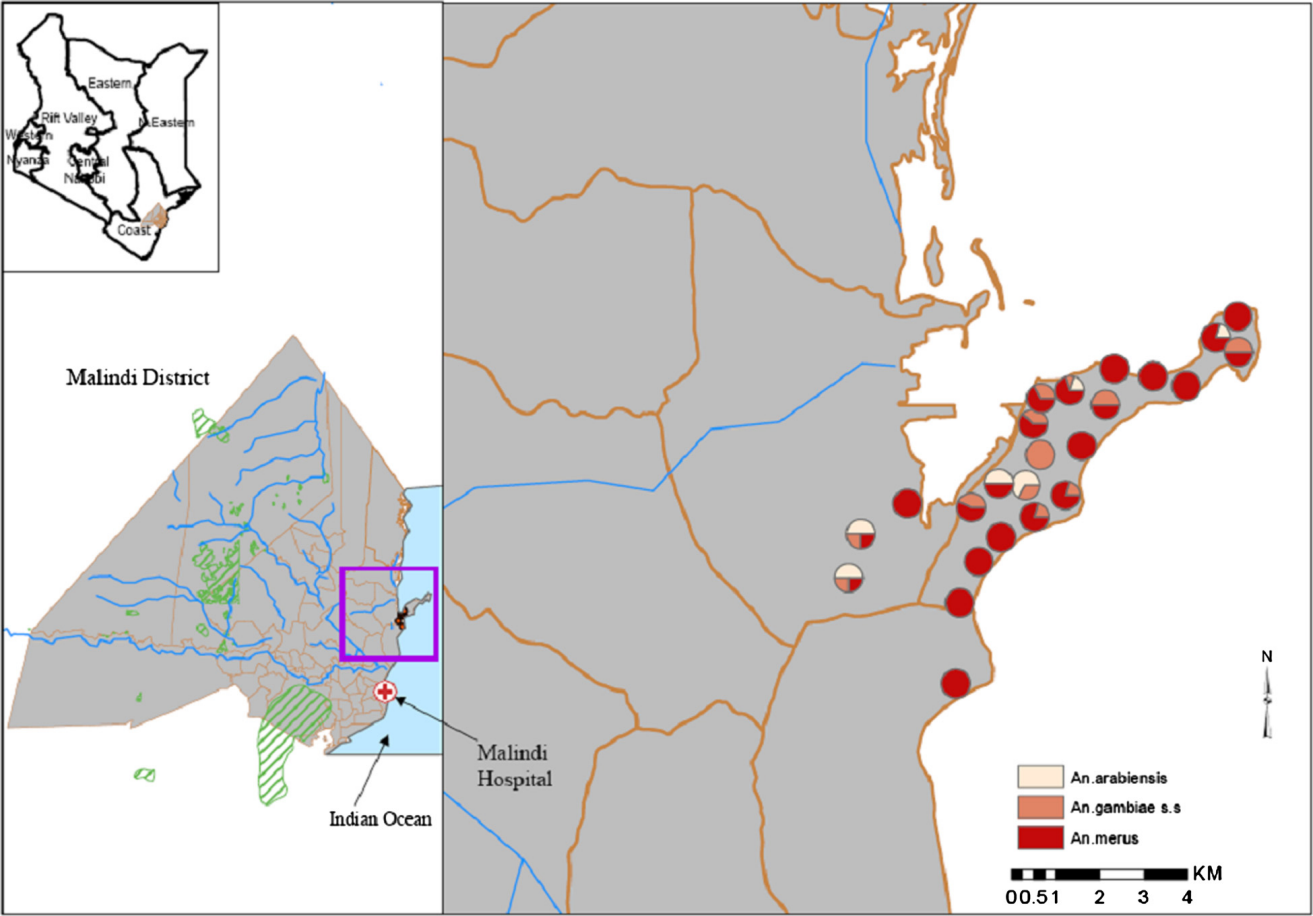
**Map of Garithe showing distribution and proportion of *An*. *merus*, *An*. *gambiae s*.*s *and *An*. *arabiensis.*** *the circles in the map represent the location (GPS coordinates) in which the *Anopheles* mosquitoes were collected.

Eighty nine percent (89%) of the 387 *An*. *gambiae* samples tested by rDNA PCR were successfully identified to species. These comprised of *An*. *merus* (77.8%), *An*. *gambiae s*.*s* (10.6%) and *An*. *arabiensis* (8%). This suggests that *An*. *merus* is the predominant species in Garithe where it occurs in sympatry with *An*. *arabiensis* and *An*. *gambiae s*.*s* although they can occupy a different ecological niche (Figure 1).

### Blood meal identification and host seeking behaviour

ELISA analysis of 83 blood fed *An*. *merus* high preference for animals as opposed to humans (Table 1). The blood meal analysis showed that a total of 31.7% (26) of the *An*. *merus* was shown to have fed on bovines, while 9.8% (9) fed on goats, 19.6% (16) was mixed feeding of bovine and goats only, 12.1% (10) fed on humans and 26.8% was unknown. Generally more blood fed *An*. *merus* were sampled indoors, 34%, as compared to those sampled outdoors, 23%. In indoor collection only 10.9% *An*. *merus* fed on humans, 24.4% fed on bovine, 15.9% fed on goat/bovine, and 4.9% fed on goat while 20.7% fed on unknown blood meal sources. In outdoor collections, 1.2% fed on humans, 7.3% fed on bovine, 3.7% cross fed on goat/bovine and 4.9% fed on goat while 6.1% fed on other sources.

**Table 1:** **Bloodmeal sources for *An*. *Merus *collected indoors (PSC and Light traps placed indoors) and outdoors (light traps placed outdoors) in Garithe, Malindi District**

			**% of blood meal sources**				
**Species**	**Collection**	**No. Tested**	**Human**	**Bovine**	**Goat/Bovine**	**Goat**	**Unknown**
*An*. *merus*	Indoors	64	12.1	24.4	15.9	4.9	20.7
	Outdoors	19	0	7.3	3.7	4.9	6.1
Total		83	12.1 (10)	31.7 (26)	19.6 (16)	9.8 (9)	26.8 (22)
*An*. *gambiae s*.*s*	Indoors	2	0	16.6	16.6	16.7	16.7
	Outdoors	4	0	0	0	16.7	16.7
Total		6	0 (0)	16.6 (1)	16.6 (1)	33.4 (2)	33.4 (2)
*An*. *arabiensis*	Indoors	2	0	25	25	0	25
	Outdoors	2	0	0	25	0	0
Total		4	0	25 (1)	50 (2)	0 (0)	25 (1)
Unknown	Indoors	4	8.3	25	16.8	16.6	16.7
	Outdoors	8	0	0	0	8.3	8.3
Total		12	8.3 (1)	25 (3)	16.8 (2)	24.9 (3)	25 (3)

None of the *An*. *gambiae s*.*s* collected indoors were observed feeding on humans, but 16.7% fed on bovine, goat/bovine, goat and other hosts. Consequently, none of the outdoor *An*. *gambiae* mosquitoes fed on humans, bovine or goat/bovine. Among the *An*. *arabiensis* collected indoors, 25% of them fed only on both bovine and goat/bovine. There was no statistical significance in feeding behaviour and blood meal sources for *An*. *merus* on human and non-human blood meals (Fishers exact test, P = 0.588) suggesting that they can feed both indoors and outdoors and are not specific in the sources of blood meal.

### Human biting rate, sporozoite rate and the Entomologic inoculation rate for *An*. *merus*

Table 2 shows the *Plasmodium falciparum* sporozoite rates for *An*. *merus*, *An*. *gambiae s*.*s* and *An*. *Arabiensis* obtained using a sporozoite ELISA method. The *Plasmodium falciparum* rate for *An*. *merus* in Garithe was 0.3%. No infections were detected in the few *An*. *gambiae s*.*s*, and *An*. *arabiensis* tested.

**Table 2:** ***Plasmodium falciparum *sporozoites for *An*. *merus*, *An*. *gambiae s*.*s*, and *An*. *arabiensis *in Garithe, Malindi**

**Species**	**No. tested**	***P*. *falciparum *ELISA%**
*An*. *merus*	301 (77.8%)	0.3
*An*. *gambiae s*.*s*	31 (8.0%)	0 (0)
*An*. *Arabiensis*	14 (3.6%)	0 (0)

The mean monthly human biting rate (HBR) and mean monthly entomological inoculation rate (EIR) for *An*. *merus* for the period September 2007 to March 2008 is shown in Table 3. The HBR for the six months ranged from 0.000 to 0.875 bites per person per month for the outdoor collections, while for the indoor collections it ranged from 0 to 1.606 bites per person per month. Overall, the HBR was highest in indoor collections during the month of September 2007 (1.606 bites per person per month), and lowest in the months of February 2008 and March 2008. The mean monthly EIR for the six month period ranged from undetectable levels to 0.018 infective bites per person. Overall, EIR for the six months was 0.003 infective bites per person both indoors and outdoors indicating that an individual would receive approximately 3 infective bites every 3 years.

**Table 3:** **Monthly mean summary of HBR and EIR of *An*. *merus *in Garithe from September 2007 to March 2008 calculated using indoor collections and outdoor collections**

***An*. *merus***	**Month**	**HBR**	**SR**	**EIR**
Outdoor collections*	September 2007	0.875	0.00	0.00
	October 2007	0.466	0.00	0.00
	November 2007	0.276	0.00	0.00
	December 2007	0.303	0.30	0.018
	January 2008	-	-	-
	February 2008	0.00	0.00	0.00
	March 2008	0.00	0.00	0.00
**Indoor collections**	September 2007	1.606	0.00	0.00
	October 2007	0.765	0.00	0.00
	November 2007	0.421	0.00	0.00
	December 2007	0.195	0.00	0.00
	January 2008	-	-	-
	February 2008	0.00	0.00	0.00
	March 2008	0.00	0.00	0.00

* was multiplied by a conversion factor of 1.605 and the HBI.

HBR-Human biting rate, SR- Sporozoite rate, EIR- Entomologic inoculation rate.

## Discussion

### Abundance of *An*. *merus* species composition

*An*. *merus* was the most abundant species sampled in Garithe, this could be because of the favourable larval habitats which are saline. Apart from being the abundant species it was found to exist in sympatry with other members of the *An*. *gambiae* complex, this shows that *An*. *merus* can co-exist with other members of the complex. These findings are similar to earlier observations made [11], which found *An*. *merus* to be one of the sibling species along the Kenyan coast.

### Vector behaviour: resting, feeding and transmission potential

There were significant differences in the number of *An*. *merus* sampled indoors and outdoors, this shows that *An*. *merus* adults rested both indoors and outdoors; the findings of this paper contrasted with those reported in Jimbo valley where it was found that *An*. *merus* rested mainly outdoors [18].

*An*. *merus* bites both indoors and outdoors and showed a tendency to feed on both human and non-human blood, most of the *An*. *merus* fed on bovine, goat or on mixed blood feeding on goat and bovine and humans suggesting that, *An*. *merus* is both zoophagic and anthropophagic. These findings were also observed in Jimbo Valley on the behavioural studies of *An*. *merus* on the Kenyan Coast [18]. From Results of the study were shown to differ from previous observations in Garithe, where it was observed that *An*. *merus* primarily fed on humans despite the availability of cows and goats [8,19], this could have been because their data was based on only blood meal analysis performed on mosquitoes collected by light traps placed indoors, therefore targeting those that feed on humans [8,19].

*An*. *merus* showed positivity for *P*. *falciparum* sporozoites with a percentage of 0.3, these findings showed that *An*. *merus* can transmit malaria parasites, as shown in studies carried out in Garithe, where the sporozoite rate of *An*. *merus* was 2.41% [11]. This demonstrates that *An*. *merus* can play a minor but important role. In Tanzania *An*. *merus* plays an important role in malaria transmission, this was shown by a study in which *An*. *merus* had a sporozoite rate of 11.6% [10]. This was also the same in Madagascar, where the role of *An*. *merus* as a malaria vector was confirmed in the case of two human-biting females, which were ELISA-positive for *Plasmodium falciparum*[9]. However, studies carried out on blood meal analysis for the Anopheline mosquitoes sampled along the Kenyan coast showed that there were no sporozoite infections found in the *An*. *merus* tested [8]. The low sporozoite rate of *An*. *merus* in Garithe could be attributed to its feeding largely on non-human blood (bovine, goat), which were abundant in every homestead sampled, this could be attributed to use of bed nets and screened windows.

### Risk of transmission

The extent of the entomologic inoculation rate is influenced by the rate at which vectors feed on humans and the sporozoite rate. In Garithe, the HBR generally was low and did not exceed previous findings in Garithe where the HBR was 2.45 infective bites per day [8], but in this study it was observed that the HBR averaged to 0.49 infective bites per day both indoors and outdoors, this in turn reduced the EIR.

Overall, the results of this study revealed the EIR for the six months was 0.003 infective bites per person both indoors and outdoors indicating that an individual would receive approximately 3 infective bites every 3 years. Thus the transmission potential for *An*. *merus* in Garithe was very low as compared to previous studies, [11]. These observations show that malaria transmission by *An*. *merus* has decreased, however, it should be taken into consideration that, it can be a significant vector at specific times of the year and that relatively high malaria parasite prevalence can occur at low or even below detectable levels of transmission [11,19,20].

## Conclusion

In conclusion, this study has shown that *An*. *merus* is the abundant species found in Garithe; this species can feed on human and non human blood, bite and rest both indoors and outdoors and have the potential to spread malaria albeit at low rates. These evidence-based findings on its resting, feeding and transmission potential will be useful for the planning of control strategies for malaria vectors.

## Competing interests

The authors declare that they have no competing interests.

## Authors’ contributions

PCK, BK, CM and JM conceived and planned the study, interpreted results and wrote the paper. PCK directed and performed the field and laboratory experiments, CM facilitated field and laboratory experiments by selecting study sites, JM assisted in obtaining local clearance from community leaders and also performed statistical analysis. All authors read and approved the final manuscript.
